# Advanced Propolis Delivery: Strategies for Improved Bioavailability and Therapeutic Efficacy

**DOI:** 10.1021/acsomega.5c12270

**Published:** 2026-05-13

**Authors:** Greta Kaspute, Tatjana Ivaskiene, Stefan Stangaciu, Urte Prentice

**Affiliations:** 226274 State Research Institute Centre for Innovative Medicine, Santariskiu St. 5, LT-08410 Vilnius, Lithuania

## Abstract

Propolis, a natural resinous product of honeybees, exhibits a wide range of biological activities, including antimicrobial, anti-inflammatory, antioxidant, and antifungal effects. However, its clinical application is limited by poor water solubility, high viscosity, and low bioavailability. To address these challenges, advanced drug delivery systems (DDS) such as nanoparticles, liposomes, hydrogels, niosomes, and self-microemulsifying systems have been developed to enhance the stability, solubility, and targeted delivery of propolis and its bioactive compounds. Lipid-based carriers and nanostructured systems have demonstrated improved mucosal penetration, cellular uptake, and controlled release profiles, leading to enhanced therapeutic efficacy against pathogens, cancer cells, and inflammatory conditions. Propolis-based formulations have shown promising applications in wound healing, oral delivery, dermatology, metabolic disorders, and hair growth. Additionally, propolis has emerged as a functional carrier matrix in DDS, contributing both structural and therapeutic benefits. Computational modeling and novel formulation strategies further support the optimization of propolis-based systems. Despite these advancements, challenges remain regarding formulation stability, safety, and clinical validation, including concerns related to allergic reactions. Future perspectives highlight the importance of integrating propolis into sustainable, multifunctional DDS and adopting holistic, personalized treatment approaches supported by artificial intelligence. Overall, propolis represents a versatile and promising component in the development of next-generation drug-delivery platforms.

## Graphical abstract

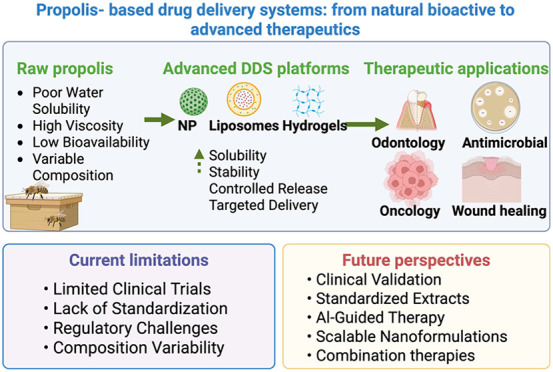

## Introduction

Propolis, a natural resinous substance produced by honeybees from special plants, is widely recognized for its therapeutic properties, including antimicrobial, anti-inflammatory, and antioxidant activities.^1^ Despite these promising biological effects, the clinical and pharmaceutical application of propolis remains significantly constrained by its poor water solubility, variable chemical composition, and limited bioavailability.^2^ These intrinsic limitations hinder its efficient absorption and reproducible therapeutic performance, thereby restricting its translation into standardized therapeutic formulations.

To address these challenges, advanced drug delivery systems (DDS) have emerged as a critical strategy to enhance the physicochemical and pharmacokinetic properties of propolis. DDS not only improve solubility and stability but also enable controlled release, targeted delivery, and enhanced cellular uptake, which are essential for maximizing therapeutic efficacy.^3^ Various delivery platforms, including nanoparticles (NP)-based systems, liposomes, hydrogels, and self-emulsifying systems, have been investigated to optimize the delivery of propolis and its bioactive constituents.^4^ For example, the self-microemulsifying DDS offers a solution for improving the absorption of poorly soluble compounds like propolis, enhancing its effectiveness in both pharmaceutical and nutraceutical applications.^2^

Among various nanocarrier systems, lipid-based formulations, such as niosomes, have demonstrated significant advantages in penetrating mucus layers, fusing with bacterial membranes, and enhancing cellular uptake in macrophages. These systems have successfully encapsulated ethanolic extracts of propolis (EEP), enabling targeted action against pathogens such as *Staphylococcus aureus* and *Candida albicans*.^5^ Furthermore, pluronic lecithin organogels, when combined with propolis, shows good bioavailability and antimicrobial efficacy. Optimized formulations incorporating lecithin and pluronic F127 demonstrated superior skin permeation and antimicrobial properties, indicating their potential for topical applications.^6^ For wound healing applications, propolis has been incorporated into electrospun nanofibers made of cellulose acetate and polycaprolactone. These nanofibrous mats demonstrates strong antioxidant and antibacterial properties.^7^ Similarly, the total flavonoid propolis extract has shown anti-inflammatory and tissue repair activities, particularly in the treatment of periodontitis, by modulating key inflammatory pathways and promoting tissue regeneration.^8^ In addition, composite wound dressings containing propolis and polycaprolactone have exhibited superior antibacterial action, antioxidant properties, and wound healing effects, making them promising candidates for advanced wound care.^9^

In addition to conventional formulation strategies, computational approaches are increasingly being applied to optimize propolis-based DDS. Techniques such as molecular docking and density functional theory have provided insights into molecular interactions between propolis constituents and carrier materials, facilitating the rational design of more efficient delivery systems. For example, studies involving poly­(vinyl alcohol) (PVA)-based systems have demonstrated enhanced anticancer activity and improved drug delivery performance *in vitro*.^10^

Despite these advancements, a systematic and critical comparison of different DDS in terms of encapsulation efficiency, release kinetics, stability, and translational feasibility remains limited. Furthermore, issues such as batch-to-batch variability of propolis, scalability of formulations, regulatory challenges, and long-term safety profiles are often underexplored, yet are crucial for clinical translation. Addressing these gaps is essential to bridge the disconnect between laboratory-scale innovations and real-world therapeutic applications.

Various extraction techniques have also been developed to improve the yield and bioactivity of propolis, including the use of polyethylene glycol (PEG)-based systems,^11^ lactic acid,^12,13^ and supercritical CO_2_ extraction.^14,15^ Extracts prepared using PEG–water or PEG–oil mixtures at elevated temperatures have demonstrated phenolic content and antimicrobial activity comparable to ethanolic extracts, whereas conventional aqueous or oil-based extractions often result in significantly lower bioactive compound recovery.^16^

Therefore, this review aims not only to summarize recent advancements in propolis-based DDS but also to critically evaluate their advantages, limitations, and translational potential. Particular emphasis is placed on comparing different delivery platforms, highlighting recent progress in clinical and preclinical studies, and identifying key challenges and future directions for the successful integration of propolis into modern therapeutic practice.

## Therapeutic Potential of Propolis

Recent studies have identified a wide range of applications for propolis and its byproducts, highlighting their significant antifungal, metabolic, and gastrointestinal benefits.^17–23^

Research on propolis extract and its byproduct has shown potent antibiofilm activity against *Candida albicans* biofilms formed on human nails, effectively permeating infected tissues without cytotoxic effects.^17^ Similarly, EEP demonstrated strong antifungal properties against *Trichophyton* species, with clinical trials reporting a 56.25% cure rate.^21^

In metabolic research, Brazilian green propolis made by bees from buds of *Bacharis dracunculifolia* has been found to ameliorate metabolic syndrome by targeting the CREB/CRTC2 transcriptional complex, a key regulator of hepatic gluconeogenesis. Artepillin C, a compound derived from propolis, inhibited CREB-CRTC2 interaction, improving insulin sensitivity and reducing lipid levels in obese mice. A novel compound, A57, exhibited even stronger inhibitory effects, suggesting CREB/CRTC2 as a promising target for antimetabolic syndrome drugs.^18^ One more study investigated a mucoadhesive thermoresponsive gel containing Brazilian green propolis extract impact on herpetic lesions. The formulation, composed of poloxamer 407 and Carbopol 934P, demonstrated favorable rheological, textural, and mucoadhesive properties, with a gelation temperature of approximately 25 °C. Propolis extract exhibited antiviral activity by inhibiting viral infection, damaging virions, and protecting cells, while the gel provided a controlled release profile, making it a promising candidate for herpes simplex virus (HSV) type 1 treatment.^65^

Additionally, a clinical trial is investigating the efficacy of propolis supplementation in treating irritable bowel syndrome (IBS). With its antioxidant, anti-inflammatory, and prebiotic properties, propolis is hypothesized to improve IBS symptoms, with outcomes focusing on severity, quality of life, and metabolic markers.^22^

Further research explored the antimicrobial and antioxidant potential of propolis byproducts, often overlooked despite their bioactive richness. These byproducts contain high levels of total phenolic and flavonoid content, demonstrating strong radical-scavenging activity, low toxicity, and antifungal effects against *Candida* species.^19,20,24^ Moreover, the combination of pink pepper and green propolis extracts enhanced antioxidant and anti-inflammatory activity through synergistic interactions. Encapsulation techniques using gum arabic and maltodextrin improved bioactive compound protection and increased radical inhibition.^25^

### Propolis as Carrier Matrix for DDS

Propolis has emerged as a promising natural carrier matrix for active drug substances, offering a dual advantage in drug delivery. Its integration into NP synthesis provides a simple, cost-effective approach to developing biocompatible and functional drug carriers. By utilizing propolis-based NP, researchers can achieve controlled drug release and targeted delivery while simultaneously harnessing the inherent biological properties of propolis. For example, liquid crystalline phase systems were prepared using PPG-5-Ceteth-20, isopropyl myristate, and water, with propolis-loaded GEL microparticles incorporated into the systems. The formulations exhibited pseudoplastic flow, thixotropy, and temperature- and composition-dependent rheological and mechanical properties, with increased surfactant concentration and propolis particles reducing the work of syringing. Drug release followed a non-Fickian pattern, showing consistent release behavior without significant differences between formulations in release times for 10, 30, and 50% of the drug.^26^ Scientists developed organogels containing ascorbic acid microparticles derived from propolis byproduct. The formulations had favorable rheological and textural properties, with good antioxidant and radical scavenging activities, and a slow release profile, particularly for the 10% microparticle formulation (F2). *In vitro* cellular studies showed no toxicity at a concentration of 63 μg/mL on HaCaT and HFF-1 cells, highlighting the potential of these organogels as a promising platform for topical drug delivery with antioxidant effects.^52^ Another study introduced microparticles made from propolis byproduct containing ascorbic acid to enhance stability. The particles were spherical with a particle size of 1654 nm, high association efficiency, and significant antioxidant capacity, demonstrated by various assays. The slow-release profile and lack of toxicity in intestinal cell lines suggest that the microparticles formulation could be a promising oral delivery system for ascorbic acid with local effects on the intestines.^46^ Another orally disintegrating films were made of gelatin and hydrolyzed collagen, incorporating ethanol extract of propolis. The films were evaluated for mechanical properties, mucoadhesion, swelling degree, *in vitro* release kinetics, stability and antimicrobial activity. Results showed that films with higher concentrations of hydrolyzed collagen were more elastic, while propolis-containing films were more resistant and exhibited improved mucoadhesion, high swelling capacity, and quick release of active compounds, with strong antimicrobial activity against *Staphylococcus aureus*, suggesting their potential for use in oral mucosa applications.^72^

The incorporation of propolis into NP synthesis improves the stability and bioavailability of therapeutic compounds, reduces toxicity, and improves cellular uptake. Additionally, natural carrier matrices like propolis contribute to sustainable and eco-friendly drug formulation strategies. Recent advancements in nanotechnology have demonstrated various methods for synthesizing propolis-based NP, including solid lipid NP (SLNs), nanostructured lipid carriers (NLCs), micellar DDS, and folic acid-conjugated NP systems. These formulations have shown significant potential in improving drug solubility, sustained release, and therapeutic efficacy, particularly in antimicrobial and anticancer applications. In this section, several examples of NP synthesis using propolis are provided to illustrate its potential in drug delivery (Table 1).

**Table 1:** Porpolis as a Source of Novel DDS Carrier Matrices

NP type	material	method	key outcome	ref.
SLNs	beeswax (BW) and propolis wax (PW)	high shear homogenization	stable NP (∼20–25 nm) with improved dispersibility and reconstitution	^27^
	Propolis		suitable for localized nasal delivery with limited permeation but enhanced bioactivity	^28^
nanostructured lipid carriers (NLCs)	BW and PW, pomegranate seed oil (PSO)	melt emulsification-ultrasonication	stable particles (71–366 nm) with tunable properties and improved lipid matrix structure	^29^
	β-sitosterol, PW, glyceryl behenate (GB), PSO	hot melt emulsification	∼100 nm particles with reduced crystallinity and improved drug incorporation	^30^
folic acid-conjugated NPs	caffeic acid phenethyl ester (CAPE)	nanoprecipitation, emulsification/self-assembly	sustained release and enhanced anticancer activity in MCF-7 cells	^31^
propolis NPs (PNP)	propolis	dynamic light scattering, scanning electron microscopy, ζ-potential.	<100 nm particles with strong antioxidant potential and safe interaction with hemoglobin	^32^

CAPE is a bioactive component derived from propolis (Table 1). It is not the primary component of propolis, but it is one of its most potent antioxidants, attributed to the caffeic acid moiety.^33^ The concentration of CAPE in propolis varies significantly depending on its geographical origin, ranging from 0 to 11 mg/gram of propolis collected in different regions of Turkey.^34^ Scientists evaluated the safety of a micellar DDS based on a newly synthesized copolymer, C12–PAGE-PG, for delivering CAPE. *In vitro* tests on HepG2 and L929 cells showed that the micelles were nontoxic, with CAPE-loaded micelles retaining their cytotoxic activity. *In vivo* studies in rats revealed no pathological changes in hematological, biochemical, or histological parameters after 14 days of treatment with either empty or CAPE-loaded micelles, indicating that C12–PAGE-PG is a safe and promising platform for drug delivery.^35^

### Improving Propolis Chemical Properties

#### Self-Nano Emulsifying DDS

Raw propolis has poor dispersibility,^2^ low bioavailability and solubility, limiting its therapeutic potential.^36^ A self-microemulsifying DDS (SNEDDS) improves EEP bioavailability with the optimal formulation comprising corn oil, Tween 80, and isopentyl alcohol in a specific ratio.^2^ Developed DDS demonstrated high drug loading capacity, stability for three months, and strong antibacterial activity against fungi, Gram-positive, and Gram-negative bacteria^2^ (Figure 1).

**Figure 1:**
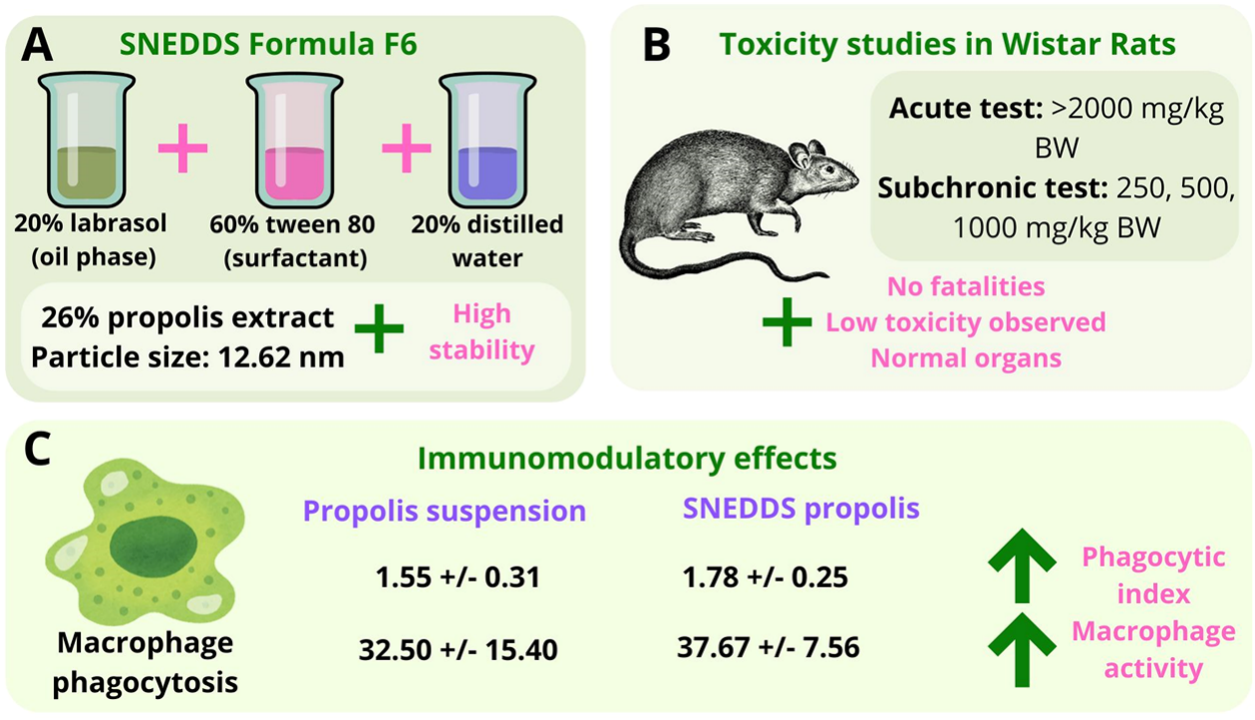
Schematic representation of SNEDDS-based propolis research. (A) Composition and characteristics of SNEDDS Formula F6, which successfully passed thermodynamic stability, dilution resistance, and accelerated stability tests.^36^ (B) Toxicity evaluation of SNEDDS propolis in Wistar rats, demonstrating low toxicity in both acute and subchronic studies.^37^ (C) Immunomodulatory effects of SNEDDS propolis, showing enhanced macrophage phagocytic activity compared to propolis suspension.^38^

#### Nanodelivery Systems of Propolis

Nanotechnology integration into novel DDS development enable to control DDS, optimize therapeutic effect while lowering possibilities of toxicity and side effects. NP combined with propolis syngergizes antibiotics, antifungals and anticancer agens, which broaden spectra of application possibilities against drug-resistant infections and cancer cells. Using biodegradable and biocompatible materials such as chitosan, SLNs, phytosomes leads to safer and nontoxic formulations development. Fpr example, scientists developed propolis-loaded SLNs as a DDS. SLNs were prepared using glyceryl monostearate, soy lecithin, Tween 80, and polyethylene glycol 400 (PEG 400) through a solvent emulsification-evaporation technique. The optimized SLNs had a mean size of 122.6 nm, with a PDI of 0.28 and a ζ-potential of −26.18 mV, demonstrating good entrapment and loading efficiencies. The study suggests that SLNs are a promising method for delivering propolis in a controlled and efficient manner.

In this section, examples of nanobased DDS containing propolis will be presented in Table 2.

**Table 2:** Examples of Nano-Propolis-Delivery Systems

type of DDS	propolis formulation	details	condition	ref
SLNs and liposomal formulations	various solvent extracts	liposomal formulations showed enhanced cytotoxicity (lowest IC50)	cancer	^39^
plasma-initiated free radical polymerization engineered carbon NPs	Russian EEP	improved antibiofilm activity via targeted release	infections	^40^
liquid crystalline system containing iron oxide magnetic NPs	propolis extract	sustained release with enhanced antifungal activity under magnetic field	periodontitis	^41^
ethylcellulose microparticles containing metronidazole and propolis	EEP+ metroinidazole	controlled release with synergistic antimicrobial effects	periodontitis	^42^
poly-(lactide-co-glycolide) acid (PLGA) NPs	sonoran propolis (SP)	enhanced anticancer activity with low toxicity	cancer	^43^
poly(n-butyl cyanoacrylate) NPs	raw propolis	BBB penetration and antifungal efficacy in vivo	infections	^44^
PVA/GEL/propolis biocompatible nanofiber patches	propolis extract	improved mechanical properties and antibacterial activity	infections	^45^
NP-based DDS made of poly(lactic-co-glycolic acid) and chitosan	EEP	sustained release with strong antiviral (HSV-2) activity	infections	^46^
niosome-based DDS with Ag85A aptamer	EEP	targeted delivery with antituberculosis activity	tuberculosis	^5^
galloyl-oligochitosan NPs (GOCNPs)	EEP	enhanced anticancer and antioxidant effects	cancer	^47^
biocompatible electrospun zein nanofibers	*Melipona quadrifasciata* propolis	controlled release and support for wound healing	wounds	^48^
phytosome-based DDS	EEP	improved solubility and skin penetration	skin-aging	^49^
lipid nanodiscs composed of 1,2-dimyristoyl-sn-glycero-3-phosphocholine (DMPC) and poly(styrene-alt-maleic acid) (PSMA)	propolis extract	enhanced aqueous solubility and low toxicity	not specified	^50^
SLNs	propolis flavonoids	sustained release with anti-inflammatory effects	skin edema	^51^
GEL microparticles	propolis extract	stable formulation with preserved antimicrobial activity	infections	^52^

Propolis is a naturally derived product produced by honeybees, and its chemical composition varies depending on the geographic region. This variation affects batch-to-batch consistency in NP formulations. Propolis is rich in bioactive compounds, which influence its solubility in water and stability. Encapsulating propolis into nanocarriers can enhance its therapeutic effectiveness. As shown in Table 2, novel propolis-based nano DDS have demonstrated promising results in many *in vitro* and *in vivo* studies. However, no clinical trials have been conducted to confirm the efficacy, pharmacokinetics, and safety of these DDS.

#### Chitosan-Based Formulations Containing Propolis

Chitosan-based DDS incorporating propolis have emerged as versatile platforms with applications across multiple biomedical fields. In odontology, these formulations enhance dentin bond strength,^53^ provide sustained antimicrobial effects,^54^ and improve drug delivery.^55^ In oncology, functionalized chitosan NP enable targeted and controlled release of propolis, increasing its anticancer potential.^56^ Additionally, antifungal chitosan films demonstrate effective activity against *Candida* species,^57^ while advanced hydrogel systems, such as chitosan-DNA and chitosan-pectin matrices, exhibit excellent biocompatibility and sustained release properties.^58^ Together, these findings highlight the broad therapeutic potential of chitosan–propolis systems (Figure 2).

**Figure 2:**
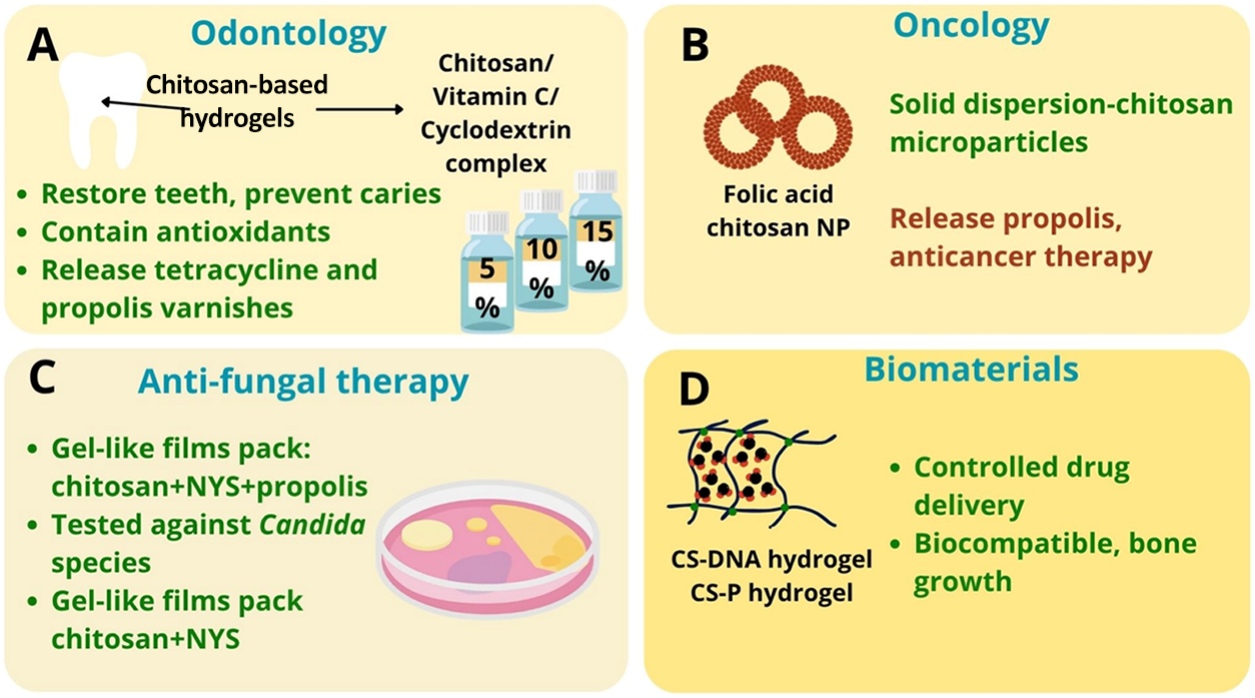
Schematic overview of chitosan-based formulations containing propolis and their biomedical applications. (A) Odontology: chitosan-based hydrogels, varnishes, and complexes (e.g., chitosan/vitamin C/cyclodextrin) enhance dentin bonding, provide controlled drug release, and exhibit antimicrobial activity for dental caries prevention. (B) Oncology: folic acid-conjugated chitosan nanoparticles and microparticle systems improve propolis bioavailability, enable controlled release, and demonstrate targeted anticancer potential. (C) Antifungal therapy: chitosan-based gel-like films incorporating propolis and/or nystatin show strong antifungal activity against *Candida* species and suitable mechanical properties for mucosal applications. (D) Biomaterials: chitosan-DNA and chitosan-pectin hydrogels act as biocompatible carriers for sustained drug delivery, supporting cell growth and tissue regeneration.

### Emulgels and Hydrogels

Hydrogels are polymeric or biopolymeric networks capable of holding large amounts of water, making them valuable for biomedical applications such as tissue engineering, wound dressings, and bioactive ingredient delivery. While synthetic polymers can be used, food and pharmaceutical applications often rely on polysaccharides and proteins like GEL, alginate, chitosan, and modified starch or cellulose. Hydrogels primarily deliver hydrophilic compounds, but when combined with emulsions, they can also encapsulate and release hydrophobic ingredients in a controlled manner^59^ (Figure 3).

**Figure 3:**
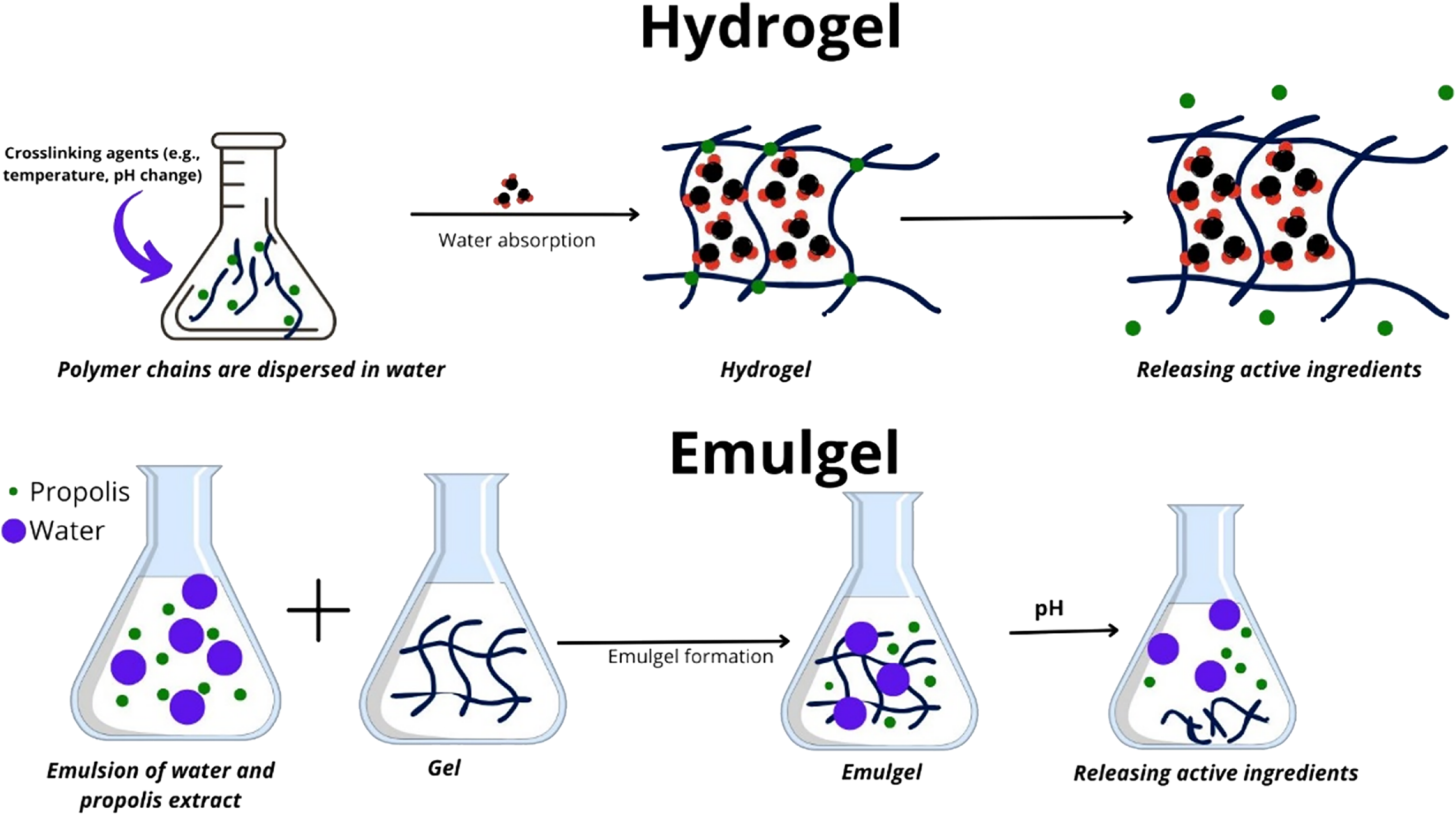
Schematic representation of hydrogels and emulgels formation and their activity as DDS.

The cryogel system was developed to control the release rate of propolis, aiming to enhance its efficacy. Propolis extracted from *Baccharis uncinella* flora in southern Brazil was incorporated at various concentrations into PVA and poly­(acrylic acid) hydrogels, with the material properties evaluated, focusing on release profiles and antibacterial activity against *Staphylococcus aureus*, *Escherichia coli*, *Salmonella typhimurium*, and *Pseudomonas putida*. The findings showed that higher propolis loading resulted in slower release rates, and the hydrogels exhibited effective antibacterial properties, suggesting their potential as bactericidal dressings for wound care.^60^ Another hydrogel was developed using mung bean protein isolate and κ-carrageenan as a copolymer for encapsulating propolis at 1 and 3% (w/v). Rheological and textural analysis showed that propolis encapsulation did not alter the hydrogel’s chewiness, adhesiveness, or hardness, while fluorescence spectroscopy confirmed electrostatic, covalent, and hydrophobic interactions in gel formation. The hydrogel demonstrated high encapsulation efficiency (>99%), a release rate of >50%, and strong antioxidant activity, making it a promising delivery system for hydrophobic bioactive compounds like propolis.^61^ A novel pH-sensitive hydrogel beads based on GEL/sodium alginate/chitosan (GEL/SA/CS) loaded with EEP were developed and their swelling behavior was examined in different pH solutions. *In vitro* release studies showed stability of the propolis-loaded beads in various pH environments mimicking the gastrointestinal tract, with the beads losing stability at pH 6.8. The results indicated that the EEP-loaded hydrogels exhibited enhanced antimicrobial activity compared to pure propolis, making them promising candidates for treating gastrointestinal diseases such as oral mucositis, gastric ulcers, and gastrointestinal cancer.^62^ One more study focused on the development and characterization of nanoemulsion-based hydrogels and organogels containing propolis and dexpanthenol, evaluating their stability, antimicrobial, and cytotoxicity properties. Characterization studies revealed successful formulations with good drug content, viscosity, spreadability, and extended drug release. Stability tests showed phase separation only at room temperature, while the antimicrobial activity was effective against *S. aureus* (hydrogel) and both *S. aureus* and *S. epidermidis* (organogel). Cytotoxicity tests demonstrated nontoxicity at low doses, indicating the potential of these systems for future use in wound and burn treatments.^63^

Emulgels, which merge the properties of emulsions and hydrogels, enhance stability, control drug release, and improve bioavailability, making them ideal for topical, ophthalmic, and functional food applications. By selecting appropriate polymers and oil phases, emulgels can be tailored for specialized drug delivery, including sustained, controlled, or burst release based on physiological triggers.^59^

Scientists explored emulgels as a delivery system for propolis and curcumin. Formulations with Carbopol 974 P (C974P) and polycarbophil (PC) demonstrated superior stability, enhanced antioxidant properties, and controlled drug release, effectively targeting *S. aureus*. These emulgels facilitated propolis and curcumin penetration into the epidermis and dermis.^64^ Another emulgel C934P and various vegetable oils was developed as a topical delivery system for propolis extract. These formulations demonstrated bioadhesive properties, prolonged drug release governed by diffusion, and effective skin permeation and retention, particularly in formulations with andiroba oil. Given propolis therapeutic potential for skin conditions, andiroba oil-based emulgels could be promising candidates for further biological and clinical studies.^65^ Scientists explored the formulation of emulgels of different polymers, such as C934P, C974P, and polycarbophil, and oils, to create a three-dimensional network for efficient delivery. The physicochemical stability of the formulations was evaluated, with passion fruit and andiroba oils showing the best results. The formulations exhibited pseudoplastic, thixotropic, and viscoelastic properties, with andiroba oil-based systems being the most stable. The presence of C934P or polycarbophil improved the mechanical and smoothness properties of the emulgels.^66^

### Liposomes

Liposomes were used as a DDS to enhance the cytotoxic effect of propolis on squamous cell carcinoma (Hep-2) cell lines. Results showed that both propolis and propolis liposomes induced apoptosis in Hep-2 cells in a dose-dependent manner, with liposomal encapsulation significantly enhancing the cytotoxic effect and apoptosis induction compared to free propolis.^67^ In another study three acetozolamide (ACZ) nanopreparations for topical application were investigated with the aim of enhancing ocular bioavailability and reducing intraocular pressure in experimental rabbits. The formulations included a liposomal phospholipid vehicle, and nanopreparations with propolis and *Punica granatum* (pomegranate). Results showed that all nanopreparations effectively reduced intraocular pressure, and Fourier transform infrared spectroscopy revealed that liposomal-ACZ and propolis formulations helped protect the optic nerve protein structure, preventing glaucoma-related changes in secondary protein structure.^68^

## Discussion

As highlighted in this review, propolis is rich in bioactive compounds, offering a wide range of therapeutic effects and applications. To overcome limitations associated with its chemical properties, encapsulating propolis in nanocarriers or innovative DDS can enhance its bioavailability, stability, and therapeutic efficacy (Table 3).

**Table 3:** Therapeutic Applications of Novel Propolis DDS

medical issue	type of DDS	purpose	results
odontology	mucoadhesive gel (Carbopol 940 and NaCMC)	periodontitis	strong mucoadhesion with sustained release and antibacterial activity.^69^
	GEL microparticles	antimicrobial delivery	effective against oral pathogens and *Candida* spp.^70^
	binary polymeric system (P407 and C934P)	endodontic application	temperature-sensitive gelation with controlled release and pulp protection potential.^71,72^
vulvovaginal candida (VVC)	mucoadhesive thermoresponsive gel (P407 and C934P)	VVC treatment	controlled release with strong antifungal activity.^73^
	mucoadhesive thermoresponsive system	*In vitro* and *in vivo* study on VVC	effective against resistant *Candida albicans*, comparable to standard therapy.^74^
	propolis extractive solution	antibiofilm study	inhibits biofilm formation and fungal growth.^75^
wounds and skin	thermosensitive bioadhesive gel (Polox/HPMC/PES)	acne treatment	enhanced release, skin permeation, and antibacterial activity.^76^
	electrospun PLGAddressing	burn wound healing	controlled release and improved healing *in vivo*.^77^
	cellulose-based films (VitC and propolis)	diabetic wound treatment	accelerated healing and reduced bacterial load.^78^
	κ-Carrageenan/β-Cyclodextrin hydrogel	antimicrobial fabric coating	sustained release with antimicrobial activity.^79^
	microneedles (PVA/PVP/P407 + propolis extract)	transdermal delivery	improved structural stability and delivery efficiency.^80^
	propolis orabase gel	denture surface study	alters surface properties affecting microbial adhesion.^81^
	patch testing	allergy assessment	identifies propolis as a potential allergen in long-term use.^82^

Despite the extensive preclinical evidence supporting propolis-based DDS, clinical translation remains limited. To date, only a few formulations, such as mucoadhesive gels for periodontitis^8,83^ and thermoresponsive vaginal gels for candidiasis,^74,75^ have reached pilot clinical studies, demonstrating favorable safety and preliminary efficacy. Other advanced systems, including electrospun wound dressings^84^ and polymeric NPs, have shown promising *in vivo* results but require further validation in human trials.^43^ The lack of large-scale, standardized clinical studies is largely due to variability in propolis composition, differences in extraction methods, and regulatory constraints, which collectively pose challenges for commercialization.^85,86^

Looking forward, future research should focus on rigorous, well-designed clinical trials to establish safety, efficacy, and dosage parameters. Standardization of propolis extracts and reproducible manufacturing protocols will be critical to meet regulatory requirements and ensure consistent therapeutic outcomes. Moreover, integrating nanocarrier technology with propolis can allow controlled release, targeted delivery, and enhanced bioavailability, addressing limitations of conventional formulations.^56,76,78,79,87–89^

The broader adoption of propolis-based therapeutics may also benefit from personalized and integrative approaches, leveraging advances in artificial intelligence and digital health tools. AI-driven platforms can optimize patient-specific dosing, select suitable DDS types, and predict potential adverse reactions based on patient profiles, thus enhancing both safety and efficacy. Additionally, the development of combination therapies, where propolis is coadministered with conventional drugs or bioactive compounds, can expand its therapeutic scope, particularly in complex diseases such as chronic infections, cancer, and diabetic wounds.

Overall, the translational and commercialization potential of propolis DDS lies in combining standardized bioactive extracts, scalable nanocarrier platforms, and rigorous clinical validation. Continued investment in clinical research, regulatory compliance, and product development will be essential for propolis-based therapeutics to transition from promising preclinical studies to clinically approved, widely accessible treatments.

## Methods

A structured literature search was conducted to identify relevant studies on propolis-based drug delivery systems (DDS) published between 1990 and 2025. The primary databases used were PubMed and Google Scholar. In PubMed, a total of 97 articles were initially retrieved, with the majority (*n* = 87) published between 2013 and 2025, reflecting the recent growth of interest in this field. After screening titles and abstracts for relevance, 77 articles were selected for further evaluation.

To capture the most recent developments, an additional targeted search was performed in Google Scholar for the years 2024–2025 using advanced search filters. This search focused on studies with titles containing “drug delivery system” and at least one mention of “propolis,” yielding 11 articles. After removal of one duplicate identified in PubMed, 10 unique records remained, of which 6 English-language articles met the inclusion criteria and were incorporated into the analysis.

**Inclusion criteria** comprised (i) original research articles published in peer-reviewed journals, (ii) studies focusing on the development, characterization, or application of propolis-based DDS, and (iii) investigations involving advanced delivery platforms such as nanoparticles, liposomes, hydrogels, or systems designed to enhance bioavailability.

**Exclusion criteria** included (i) review articles, (ii) conference abstracts, (iii) non-English publications, and (iv) studies lacking sufficient experimental detail or not directly related to drug delivery applications of propolis.

Following selection, full-text articles were critically evaluated with emphasis on formulation strategies, encapsulation efficiency, release behavior, therapeutic outcomes, and potential for clinical translation. This approach enabled a focused and comparative assessment of current advances in propolis-based DDS.

## Conclusion

Integration of propolis-derived materials into innovative DDS enhances the bioavailability and controlled release of active substances in targeted therapeutic areas. Future studies should investigate the stability, safety, and bioavailability of propolis in nanoformulations. Exploring synergistic effects with other therapeutic agents could further expand its potential in combination therapies. Additionally, developing eco-friendly and sustainable production methods for propolis-based DDS will be essential in today’s world.

## Data Availability

Data sharing does not apply to this article as no data sets were generated.
